# Trans-Arterial Embolization for Liver Metastases of Gastroenteropancreatic Neuroendocrine Tumors: Response Indicates Survival Benefit?

**DOI:** 10.3390/cancers17020309

**Published:** 2025-01-19

**Authors:** Luohai Chen, Dequan Yang, Yueriguli Yusufu, Haikuan Liu, Man Liu, Yuan Lin, Yanji Luo, Qiao He, Minhu Chen, Zhirong Zeng, Ning Zhang, Yu Wang

**Affiliations:** 1 Department of Gastroenterology, The First Affiliated Hospital, Sun Yat-sen University, Guangzhou 510080, China; chenlh76@mail.sysu.edu.cn (L.C.); yusufu@mail2.sysu.edu.cn (Y.Y.); lium75@mail.sysu.edu.cn (M.L.); chenminhu@mail.sysu.edu.cn (M.C.); zengzhirong@mail.sysu.edu.cn (Z.Z.); 2 Department of Interventional Oncology, The First Affiliated Hospital, Sun Yat-sen University, Guangzhou 510080, China; yangdq23@mail2.sysu.edu.cn (D.Y.); liuhk5@mail2.sysu.edu.cn (H.L.); 3 Department of Pathology, The First Affiliated Hospital, Sun Yat-sen University, Guangzhou 510080, China; liny36@mail.sysu.edu.cn; 4 Department of Radiology, The First Affiliated Hospital, Sun Yat-sen University, Guangzhou 510080, China; luoyj26@mail.sysu.edu.cn; 5 Department of Nuclear Medicine, The First Affiliated Hospital, Sun Yat-sen University, Guangzhou 510080, China; heqiao6@mail.sysu.edu.cn

**Keywords:** gastroenteropancreatic neuroendocrine tumors, trans-arterial embolization, overall survival

## Abstract

Objective: To determine the impact of trans-arterial embolization (TAE) on overall survival (OS) in patients with liver metastases from gastroenteropancreatic neuroendocrine tumors (LM-GEP-NETs) and to identify factors that may influence tumor response to TAE treatment. Methods: This study included patients with histologically and radiologically confirmed LM-GEP-NETs who received TAE treatment at The First Affiliated Hospital, Sun Yat-sen University, between November 2016 and January 2023. Imaging responses were assessed using Response Evaluation Criteria in Solid Tumors (RECIST) 1.1 and modified RECIST (mRECIST) criteria. Tumor response was defined as complete or partial remission. Results: In total, 267 patients with LM-GEP-NETs were included. Patients with liver tumor burdens <25%, 25–50%, and ≥50% had progressively worse OS (*p* < 0.005). According to the RECIST criteria, 65.9% of patients exhibited tumor responses. Using the mRECIST criteria, 77.5% of patients showed tumor responses. Survival analyses with log-rank tests indicated that patients with tumor responses assessed using either the RECIST or mRECIST criteria had significantly better OS (*p* = 0.015 and *p* = 0.023, respectively). Further logistic regression analyses showed that early TAE (within 4 months after diagnosis of liver metastases) was associated with tumor responses assessed using RECIST or mRECIST. These results were further verified using propensity score matching and inverse probability treatment weighting adjusted datasets. Conclusions: A higher liver tumor burden was associated with poorer OS in patients with LM-GEP-NETs. Tumor response after TAE indicates survival benefits. Early TAE (within 4 months of diagnosis) was associated with better treatment responses.

## Simple Summary

Liver metastases of gastroenteropancreatic neuroendocrine tumors (LM-GEP-NETs) may affect treatment response and patient survival. Trans-arterial embolization (TAE) is a widely used liver-directed treatment for diffused LM-GEP-NETs. In this study, we demonstrated that a higher liver tumor burden was associated with poorer overall survival (OS) in patients with LM-GEP-NETs. Additionally, tumor response after TAE was associated with better OS. Further analyses indicated that early TAE (within 4 months of diagnosis) was associated with better treatment responses.

## 1. Introduction

Gastroenteropancreatic neuroendocrine tumors (GEP-NETs) are a rare group of tumors derived from the diffuse endocrine cells of the digestive system. Although GEP-NETs are highly heterogeneous, they share a common feature: liver metastases (LM) occur in up to 90% of patients with advanced GEP-NETs [1]. Additionally, LM of GEP-NETs (LM-GEP-NETs) may affect treatment response and patient survival. In the PROMID study, liver tumor burden was associated with an unfavorable time to progression or tumor-related death in patients receiving octreotide long-acting release (LAR) treatment [2]. Our previous study indicated that a high liver tumor burden is associated with poor overall survival (OS) and poor progression-free survival in patients with GEP-NETs treated with octreotide LAR [3]. Thus, more attention should be paid to LM-GEP-NETs to help improve patient survival.

Liver-directed treatments for diffused LM-GEP-NETs, including trans-arterial embolization (TAE) and trans-arterial chemotherapy embolization (TACE), are widely used for these patients. Several studies, including ours, have shown that complete remission (CR) or partial remission (PR) of LM-GEP-NETs after TAE/TACE treatment can be observed in 20–100% of patients [4,5]. In addition, TAE/TACE is well tolerated in patients with LM-GEP-NETs, with grade 1/2 abdominal pain and fever being the most common adverse effects, which typically resolve within one week [6]. However, previous studies were limited by their small sample sizes, and the impact of tumor response in LM-GEP-NETs following TAE/TACE treatment on patient OS still needs further validation [7,8]. A recent study showed that 50% of patients with LM from NETs achieved partial responses to LM after TACE, while patients with PR had better OS [9]. Previous studies have suggested that TACE does not provide better efficacy and might cause more adverse effects than TAE [10]. Therefore, it is critical to investigate the role of TAE and the effects of post-TAE PR/CR on OS in a large cohort of patients with LM-GEP-NETs.

In addition, if tumor response after TAE can help prolong patients’ OS, another important question is how to improve tumor response. However, the factors associated with tumor response rates to TAE for LM-GEP-NETs have rarely been studied. Furthermore, the optimal timing for performing TAE is still not well understood. As a result, in the real world, patients may undergo TAE at different stages of the disease. Whether early TAE treatment can provide a better tumor response has not yet been explored. Therefore, this study aimed to determine whether tumor response after TAE can lead to prolonged OS in patients with LM-GEP-NETs and to identify factors associated with tumor response to TAE treatment, including the latency to TAE, using a large retrospective cohort.

## 2. Methods

### 2.1. Patients

This study included patients with pathologically and radiologically confirmed well-differentiated LM-GEP-NETs treated with TAE between November 2016 and January 2023 at The First Affiliated Hospital, Sun Yat-sen University. Patients treated with TAE/TACE before admission to our hospital were excluded due to the unavailability of complete baseline data on their clinicopathological characteristics. The indication for TAE at our center is as follows: patients with disseminated LM-GEP-NETs that cannot be completely resected or treated with surgery or ablation, and whose primary tumor burden is in the liver. Contraindications for TAE include previous pancreaticoduodenectomy, liver failure, renal failure, iodine allergy, and others.

Clinicopathological information, including age, sex, primary site, tumor grade, surgery before TAE, functionality, liver tumor burden, and concurrent systemic treatments, was collected and reviewed. Sites of metastases were confirmed by combining results from routine PET/CT, CT, and/or MRI scans. The dates of diagnosis of LM, first TAE cycles, last follow-up, and death were also collected. This study was approved by the Ethics Committee of The First Affiliated Hospital, Sun Yat-sen University, and the requirement for informed consent was waived.

### 2.2. TAE Procedure and Tumor Response Assessment

Within one month before the initial TAE treatment, enhanced CT imaging was performed to provide baseline data for subsequent treatments. All CT images and liver tumor burdens were retrospectively reviewed and assessed by two radiologists with three and 11 years of experience. TAE was performed under local anesthesia in the interventional radiology suite. Using the Seldinger technique, a 5F catheter was inserted into the celiac trunk for digital subtraction angiography. A microcatheter was selectively advanced into the feeding arteries of the tumor. Embolization was first carried out using 40–120 μm embosphere (Merit Medical, South Jordan, UT, USA), followed by 100–300 μm polyvinyl alcohol (Cook, Bloomington, IN, USA). The endpoint of embolization was defined as the absence of contrast medium clearing at the tip of the microcatheter within two to five cardiac cycles.

CT imaging was repeated 4–6 weeks after TAE to assess the effectiveness of the embolization for LM using the response evaluation criteria in solid tumors (RECIST) 1.1 and modified RECIST (mRECIST) criteria [11,12]. Embolization efficacy was classified as complete remission (CR), partial remission (PR), stable disease (SD), or progressive disease (PD). CR and PR were further defined as tumor responses. Based on these imaging follow-up results, additional embolization may be performed, or TAE treatment might be discontinued. In general, three procedures of TAE would be performed. TAE would be halted before completing three procedures if PD was identified on repeated CT scans. For patients with a very high liver tumor burden, more than three procedures of TAE might be performed unless PD occurred before the completion of three procedures, to avoid severe adverse effects caused by insufficient liver blood supply or extensive tumor necrosis within a short period of time.

### 2.3. CT Protocols and Assessments

Abdominal multi-slice CT scans (Aquilion 64, Toshiba Medical Systems, Tokyo, Japan) were performed in accordance with the standard scanning protocol (tube voltage, 120 kV; tube current, 200 mA; beam collimation, 6 × 0.5 mm; slice thickness, 1 mm; slice increments, 0.5 mm). Iodinated contrast (Ultravist 300, Bayer Schering, Berlin, Germany) was injected into antecubital vein of patients at a flow rate of 3–4 mL/s using an automatic injector with a volume of 1.5 mL/kg, after the nonenhanced phase scan. Then, a 40-mL bolus of saline solution was infused. Arterial and portal venous phases were acquired at 35 and 65 seconds after the initiation of iodinated contrast medium injection, respectively. Two radiologists with 3+ and 10+ years of experience assessed tumor response according to the RECIST 1.1 and mRECIST criteria within two days after each scan. Since patients’ OS data were obtained afterward, the tumor response assessments were independent of the patients’ outcomes.

### 2.4. Statistical Analysis

Overall survival (OS) was calculated from the time of diagnosis of LM from GEP-NETs to the time of death or last follow-up. Kaplan-Meier analyses with log-rank tests were used to compare OS among groups. A univariate Cox proportional hazards model was applied to identify risk factors for OS, and those with a *p* value < 0.05 were further analyzed using a multivariate Cox proportional hazards model, adjusted for age, sex, primary site, tumor grade, functionality, and liver tumor burden. Classification and regression tree analysis was used to determine the optimal cutoff value for latency to TAE for a better treatment response. Univariate and multivariate logistic regression analyses (adjusted for age, sex, primary site, tumor grade, functionality, and liver tumor burden) were performed to identify factors associated with tumor response, including CR and PR. Propensity score matching and inverse probability of treatment weighting (IPTW) were further used to validate the role of early TAE treatment in tumor response. All analyses were performed using the R statistical software (version 4.3.1; R Foundation for Statistical Computing, Vienna, Austria). Statistical significance was defined as a two-sided *p* value < 0.05.

## 3. Results

### 3.1. Basic Demographics of Patients with LM-GEP-NETs

A total of 267 patients with LM-GEP-NETs were included in this study (Table 1). The mean age of the patients was 53 years. Women were slightly more common among the patients, accounting for 54.7% of the cohort. The most common primary tumor sites were the pancreas (55.1%) and the rectum (27.7%). WHO tumor grades 1, 2, and 3 were observed in 12.7%, 80.1%, and 7.1% of the patients, respectively. Most patients (85.4%) had nonfunctional tumors. Liver tumor burdens of <25%, 25–50%, and ≥50% were found in 45.3%, 21.0%, and 33.7% of patients, respectively. Approximately 33% of patients had extrahepatic metastases. Primary tumor resection before TAE was performed in 56.9% of the patients, while partial liver resection and liver-directed ablation before TAE were performed in 16.1% and 9.7% of patients, respectively. 17.0%, 30.3%, 41.6%, and 11.2% of patients underwent one, two, three, and more than four TAE procedures, respectively. Most patients (79.0%) received somatostatin analogs during the TAE treatment. The average latency to TAE was 3 months.

### 3.2. Survival Analysis of Patients with LM-GEP-NETs Treated with TAE

**Figure 1:**
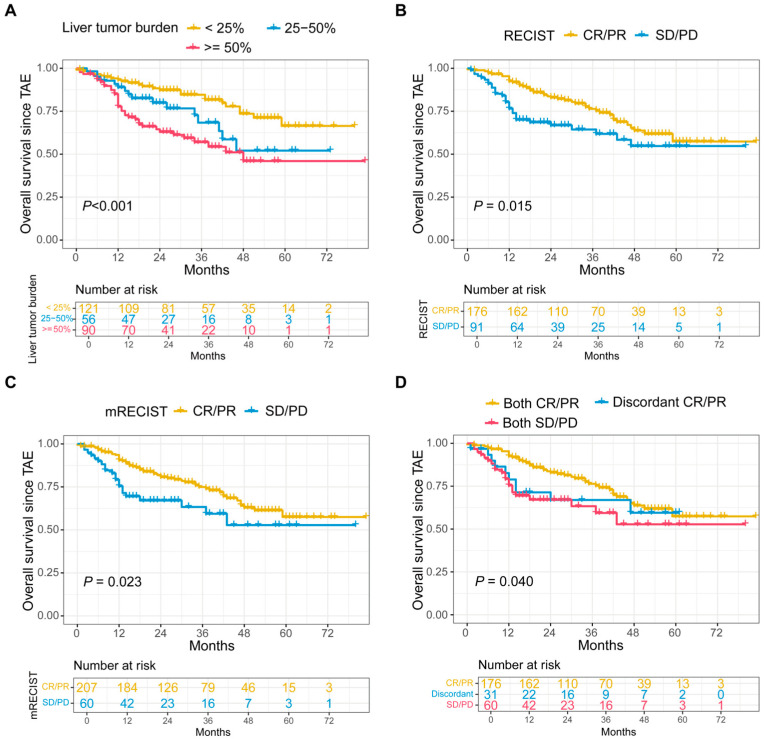
(**A**) Survival analysis of patient with different liver tumor burdens. (**B**) Comparison of survival of patients with different tumor response after TAE treatments assessed using RECIST criteria. (**C**) Comparison of survival of patients with different tumor response after TAE treatments assessed using mRECIST criteria. (**D**) Comparison of survival of patients with different tumor response after TAE treatments assessed using RECIST and mRECIST criteria. Both CR/PR and both SD/PD means consistent CR/PR and SD/PD, respectively, using these two criteria. While discordant CR/PR means CR/PR using mRECIST criteria but SD/PD using RECIST criteria.

We first analyzed whether liver tumor burden affected OS in patients with LM-GEP-NETs. As expected, patients with liver tumor burdens of <25%, 25–50%, and ≥50% had progressively worse OS (*p* < 0.005, Figure 1A). Next, we examined whether TAE could reduce liver tumor burden and improve OS. According to the RECIST criteria, CR, PR, SD, and PD as the best responses were observed in five patients (1.9%), 171 (64.0%), 90 (33.7%), and one patient (0.4%). Thus, 65.9% of patients exhibited tumor responses, while 34.1% did not. Among patients who received only one, two, three, and >4 procedures, rate of RECIST CR/PR were 33.3%, 70.4%, 76.6%, and 63.3%.

Using the mRECIST criteria, CR, PR, SD, and PD as the best responses were observed in 38 (14.2%), 169 (63.3%), 59 (22.1%), and 1 (0.4%) patient. Overall, 77.5% of patients showed tumor responses, while 34.1% did not. Among patients who received only one, two, three, and >4 procedures, the rates of mRECIST CR/PR were 57.8%, 80.2%, 86.5%, and 66.7%.

Further survival analyses indicated that patients with tumor responses assessed using the RECIST criteria had significantly better OS compared to those without responses (*p* = 0.015; Figure 1B). Univariate (hazard ratio [HR], 0.570; 95% confidence interval [CI], 0.388–0.993; *p* = 0.016) and adjusted Cox analyses (HR, 0.621; 95% CI, 0.360–0.901; *p* = 0.047) indicated that tumor responses of CR/PR assessed by RECIST criteria were significantly associated with reduced death risk. Similarly, patients with tumor responses assessed using the mRECIST criteria had significantly better OS compared to those without responses (*p* = 0.023; Figure 1C). Univariate (HR, 0.560; 95% CI, 0.338–0.928; *p* = 0.025) and adjusted Cox analyses (HR, 0.464; 95% CI, 0.268–0.805; *p* = 0.006) also showed that tumor responses of CR/PR assessed by mRECIST criteria were associated with reduced death risk.

**Figure 2:**
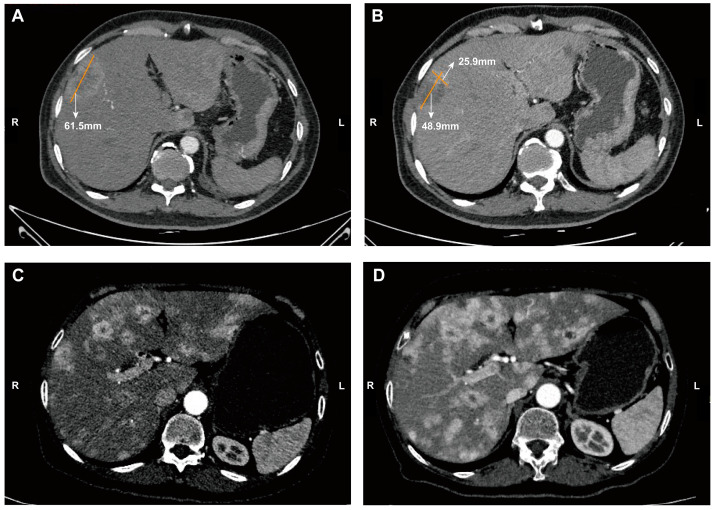
(**A**) Baseline CT image from a 56-year-old male patient with liver metastases of a WHO Grade 3 pancreatic neuroendocrine tumor. The longest diameter of one target lesion of liver metastasis was 61.5 mm. (**B**) After two procedures of TAE treatment, the longest diameter of this target lesion decreased to 48.9 mm. The longest diameter of the enhanced tumor region decreased to 25.9 mm. The general tumor response assessment for this patient was SD using RECIST criteria but partial response using mRECIST criteria. (**C**) Baseline CT image of liver metastases from a 66-year-old female patient with liver metastases with a WHO Grade 2 pancreatic neuroendocrine tumor. (**D**) After one procedure of TAE treatment, the patient had PD.

We then investigated whether the RECIST or mRECIST criteria were more appropriate for evaluating tumor response to TAE treatments. The best tumor responses in 60 patients were CR/PR when assessed using mRECIST criteria but SD/PD when assessed using RECIST criteria. Tumor responses for the remaining patients were identical using both criteria. Survival analysis indicated that the OS of patients with discordant response results was more similar to that of patients with consistent SD/PD using both criteria (Figure 1D and Figure 2). Additionally, Harrell’s c-index was significantly higher when using RECIST criteria (0.596, 95% CI: 0.537–0.655) compared to mRECIST criteria (0.572, 95% CI: 0.515–0.629; *p* < 0.001).

### 3.3. Identification of Factors Associated with Tumor Response After TAE Treatments

**Figure 3:**
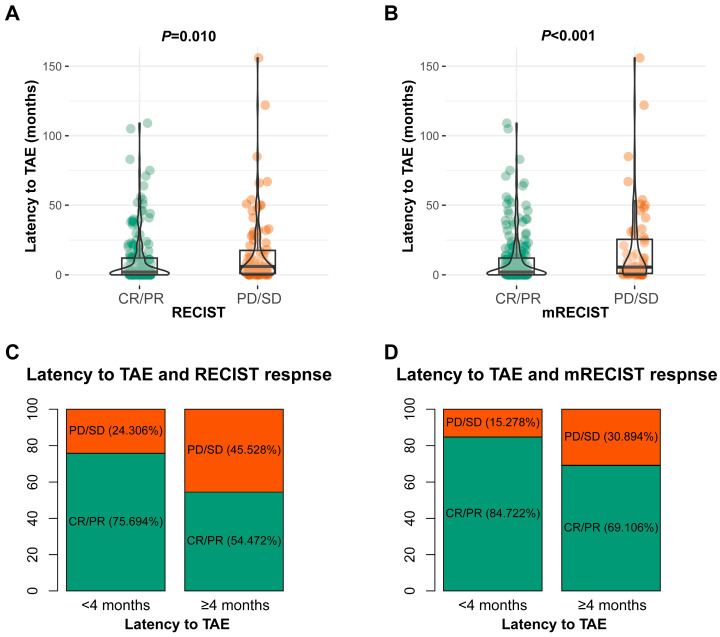
(**A**) Relationship between tumor response assessed using RECIST criteria and latency to TAE. (**B**) Relationship between tumor response assessed using mRECIST criteria and latency to TAE. (**C**) Comparison of tumor response rates assessed using RECIST criteria between patients with early (<4 months) and late (≥4 months) TAE treatments. (**D**) Comparison of tumor response rates assessed using mRECIST criteria between patients with early (<4 months) and late (≥4 months) TAE treatments.

First, we assessed the effect of latency to TAE from the diagnosis of LM on tumor responses, as this factor might influence treatment decisions in the future. Patients with tumor responses of CR/PR had significantly shorter latencies to TAE (Figure 3A,B), indicating the potential effect of early TAE treatment in improving tumor responses. Classification and regression tree analysis identified the optimal cutoff value for latency to TAE as 4 months. Patients treated with TAE for <4 months after the diagnosis of LM-GEP-NETs had significantly higher rates of CR/PR (Figure 3C,D).

Univariate and adjusted logistic regression analyses were performed to identify other factors associated with tumor responses and the independent role of TAE latency in tumor responses (Table 2). A latency of <4 months was consistently associated with better tumor responses of CR/PR.

### 3.4. Analysis of the Role of Early TAE in Tumor Response Using Propensity Score and IPTW

To further validate the impact of early TAE on tumor response, latency <4 months was defined as early TAE, while latency ≥4 months was defined as late TAE. Propensity score matching and IPTW were used. Factors including age, sex, primary site, tumor grade, functionality, primary tumor resection, partial liver resection before TAE, liver-directed ablation before TAE, and liver tumor burden were matched or adjusted using propensity score or IPTW (Table 3).

In the propensity score-matched dataset, the tumor response rate assessed using the RECIST criteria was significantly higher in patients who received early TAE treatment (92/123, 74.8%) compared to those who received late TAE treatment (67/123, 54.5%; *p* = 0.001). Both univariate (odds ratio [OR], 2.481; 95% CI, 1.454–4.291; *p* < 0.001) and adjusted logistic regression analyses (OR, 2.540; 95% CI, 1.464–4.481; *p* = 0.001) indicated that early TAE was associated with better tumor response. Similar results were observed using the mRECIST criteria; early TAE treatment (104/123, 84.6%) showed a significantly better tumor response rate than late TAE treatment (85/123, 69.1%; *p* = 0.007). Both univariate (OR, 2.447; 95% CI, 1.330–4.628; *p* = 0.005) and adjusted logistic regression analyses (OR, 2.636; 95% CI, 1.393–5.151; *p* = 0.004) indicated that early TAE was associated with better tumor response.

In the IPTW-adjusted dataset, univariate (OR, 1.219; 95% CI, 1.077–1.379; *p* = 0.002) and adjusted logistic regression analyses (OR, 1.219; 95% CI, 1.084–1.371; *p* = 0.001) showed that early TAE was also associated with improved tumor response assessed using RECIST criteria. Similar results were observed for the mRECIST criteria. Univariate (OR, 1.167; 95% CI, 1.043–1.305; *p* = 0.007) and adjusted logistic regression analyses (OR, 1.164; 95% CI, 1.050–1.290; *p* = 0.004) demonstrated that early TAE was associated with better tumor response.

## 4. Discussion

In this study, we investigated the survival benefits of TAE treatment in patients with LM-GEP-NETs. Unlike previous studies, we examined the relationship between tumor response after TAE treatment and OS using a large dataset. Additionally, we explored potential clinicopathological factors associated with TAE treatment response and provided a comprehensive analysis of the effect of early TAE on improving tumor response.

Liver tumor burden has been identified in several studies as a significant prognostic factor affecting the efficacy of medical treatments and patients’ OS [3,4,13]. Consistent with previous findings, our study also assessed the prognostic value of liver tumor burden, showing that patients with a higher liver tumor burden had significantly worse OS. Recently, peptide receptor radionuclide therapy (PRRT) with ^177^Lu-DOTATATE demonstrated unprecedentedly good tumor responses in patients with GEP-NETs (as shown in the NETTER-1 and NETTER-2 trials) [14,15]. However, similar to other medical treatments, a higher liver tumor burden was associated with shorter PFS in patients receiving PRRT [16]. Liver-directed local treatments [17], such as TAE, are usually applied in combination with systemic treatments. Previous studies have indicated that TAE helps reduce liver tumor burden and may play a role in further improving the efficacy and PFS of systemic therapies like PRRT.

The association between tumor response after TAE treatments and patient prognosis with LM-GEP-NETs was analyzed in several previous studies [7,8]. Jessica Assouline and colleagues conducted a retrospective study including 119 patients with neuroendocrine liver metastases who received TAE or TACE [7]. They found that decreased liver-enhancing tumor burden after TAE/TACE was independently associated with better OS. Similar results were found in a recent study that showed patients with LM from NETs who achieved PR after TACE had better OS than their counterparts [9]. Unlike previous studies, our study did not include patients who received TACE, and we analyzed the survival benefits of tumor response after TAE treatment in patients with LM-GEP-NETs using a large dataset. The sufficient sample size in our study provides more compelling evidence that patients who show tumor responses after TAE have improved OS. Thus, decreasing liver tumor burden through TAE is associated with survival benefits. Future studies are needed to develop improved liver-directed treatments to increase tumor response rates. Additionally, our results suggest that the tumor responses assessed using the RECIST or mRECIST criteria are suitable parameters for evaluating the efficacy of TAE. We further compared RECIST and mRECIST to assess which criteria might be more appropriate for evaluating tumor response in patients with LM-GEP-NETs who received TAE treatment. More CR/PR responses were observed when assessed using the mRECIST criteria. However, the OS of patients who had CR/PR using mRECIST but SD/PD using RECIST was more similar to those with SD/PD assessed by both criteria. Considering that RECIST criteria also had a higher Harrell’s c-index, RECIST criteria may be more suitable for evaluating tumor response in patients receiving TAE treatments.

Given that tumor response after TAE treatment provides survival benefits to patients with LM-GEP-NETs, it is important to identify the factors associated with treatment response, especially factors that can be managed in clinical practice. In this study, our findings revealed that TAE latency correlated with tumor response. Patients who underwent early TAE had significantly higher CR and PR rates. These results were further validated using two methods, propensity score (PS) and inverse probability of treatment weighting (IPTW), which helped adjust for confounding bias [18]. Therefore, early TAE treatment is essential for patients with LM-GEP-NETs as it could improve tumor response and provide survival benefits to patients. These results also indicate that early liver-directed treatment may be important for patients with LM-GEP-NETs; however, this requires further investigation.

In this study, we observed that patients who underwent one, two, or three TAE procedures had progressively higher rates of CR/PR. This phenomenon may primarily be due to the TAE protocol used at our center. Patients with better tumor responses were more likely to undergo additional TAE procedures unless they had achieved CR or completed three TAE procedures. On the other hand, patients who received more than three TAE procedures had lower rates of CR/PR. This could be attributed to the high liver tumor burden in these patients. To avoid severe adverse effects caused by insufficient liver blood supply or excessive tumor necrosis within a short period [19,20], we typically opted for a staged approach to arterial embolization. As a result, additional procedures were required. This finding also suggests that patients with very high liver tumor burdens may experience a diminished tumor response to TAE.

This study was limited by its retrospective design. However, given that GEP-NETs are a rare group of tumors, conducting randomized controlled trials is challenging. Our study confirmed the impact of tumor response after TAE treatment on patient OS using a large dataset and provided valuable evidence for the critical role of early TAE in LM-GEP-NETs, a topic that has rarely been reported.

## 5. Conclusions

In conclusion, liver tumor burden is associated with OS in patients with LM-GEP-NETs. The objective tumor response after TAE is associated with survival benefits, as indicated by prolonged OS. Latency to TAE is related to the tumor response rate, with early TAE (<4 months) playing a significant role in improving the tumor response to TAE treatment.

## Figures and Tables

**Table 1:** Basic characteristics of patients with LM-GEP-NETs.

Characteristics	N (%) or Median
Age, years	53
Sex	
Male	121 (45.3)
Female	146 (54.7)
Primary sites	
Pancreas	147 (55.1)
Stomach	14 (5.2)
Small intestine	32 (12)
Rectum	74 (27.7)
Ki-67 index, %	8
Grade	
Grade 1	34 (12.7)
Grade 2	214 (80.1)
Grade 3	19 (7.1)
Functionality	
Functional	39 (14.6)
Non-functional	228 (85.4)
Liver tumor burden	
<25%	121 (45.3)
25–50%	56 (21.0)
≥50%	90 (33.7)
Extrahepatic metastases	
Yes	178 (66.7)
No	89 (33.3)
Primary resected	
Yes	115 (43.1)
No	152 (56.9)
Partial liver resection	
Yes	43 (16.1)
No	224 (83.9)
Liver-directed ablation before ^1^	
Yes	26 (9.7)
No	241 (90.3)
Concurrent systemic treatment	
SSAs	211 (79.0)
TKIs	10 (3.7)
Everolimus	5 (1.9)
Chemotherapy	30 (11.2)
Combinations	5 (1.9)
No	6 (2.2)
Latency to TAE, months	3

^1^ Liver-directed ablations including radiofrequency ablations (23 patients), microwave ablations (2 patients), and cryoablations (1 patient).

**Table 2:** Logistic regression analysis of characteristics associated with complete or partial remission of LM-GEP-NETs after TAE.

CR/PR (RECIST)						CR/PR (mRECIST)		
	Univariate Analysis		Adjusted Analysis *		Univariate Analysis		Adjusted Analysis *	
	OR (95%CI)	*p*	OR (95%CI)	*p*	OR (95%CI)	*p*	OR (95%CI)	*p*
Age, years	0.988 (0.968–1.009)	0.258			1.001 (0.978–1.024)	0.962		
Sex								
Female	1				1			
Male	0.777 (0.467–1.291)	0.330			0.855 (0.480–1.525)	0.594		
Primary sites								
Pancreas	1		1		1		1	
Stomach	2.529 (0.752–11.527)	0.168	2.491 (0.663–1.288)	0.215	2.563 (0.663–16.910)	0.230	2.518 (0.626–16.960)	0.249
Small intestine	3.724 (1.463–11.471)	0.011	2.605 (0.981–8.256)	0.073	2.990 (1.092–10.542)	0.052	2.842 (1.014–10.163)	0.069
Rectum	1.529 (0.853–2.797)	0.160	1.186 (0.632–2.258)	0.598	2.734 (1.330–6.098)	0.009	3.167 (1.445–7.469)	0.006
Grade								
Grade 1	1				1			
Grade 2	0.984 (0.439–2.091)	0.967			1.036 (0.416–2.346)	0.935		
Grade 3	0.430 (0.133–1.353)	0.152			1.641 (0.406–8.328)	0.508		
Functionality								
Non-functional	1				1			
Functional	1.040 (0.514–2.193)	0.915			0.600 (0.288–1.308)	0.182		
Liver tumor burden								
<25%	1				1			
25–50%	0.762 (0.391–1.505)	0.428			0.525 (0.249–1.118)	0.091		
≥50%	0.666 (0.373–1.184)	0.166			0.612 (0.312–1.193)	0.149		
Extrahepatic								
metastases								
No	1				1			
Yes	0.776 (0.445–1.330)	0.362			0.821 (0.433–1.511)	0.534		
Primary resected								
No	1				1			
Yes	0.947 (0.569–1.581)	0.834			0.904 (0.507–1.620)	0.732		
Partial liver resection								
No	1				1			
Yes	0.755 (0.388–1.498)	0.411			0.535 (0.265–1.119)	0.087		
Liver-directed ablation before								
No	1				1			
Yes	0.569 (0.251–1.306)	0.176			0.507 (0.218–1.252)	0.124		
Concurrent systemic treatment								
SSAs	1		1		1		1	
TKIs	0.102 (0.015–0.420)	0.005	0.118 (0.017–0.514)	0.010	0.351 (0.096–1.426)	0.117	0.394 (0.091–1.790)	0.208
Everolimus	0.610 (0.099–4.716)	0.593	0.576 (0.088–4.659)	0.564	0.351 (0.056–2.729)	0.260	0.648 (0.090–5.682)	0.667
Chemotherapy	0.702 (0.320–1.608)	0.387	0.727 (0.317–1.727)	0.457	0.936 (0.379–2.659)	0.892	0.809 (0.259–2.880)	0.727
Combinations	0.271 (0.035–1.674)	0.158	0.407 (0.051–2.596)	0.340	0.156 (0.020–0.970)	0.046	0.150 (0.018–1.012)	0.051
No	<0.001 (NA-1.558 × 10^33^)	0.986	<0.001 (NA-1.514 × 10^32^)	0.986	0.047 (0.002–0.300)	0.006	0.062 (0.003–0.430)	0.015
Latency to TAE, months								
≥4	1		1		1		1	
<4	2.602 (1.554–4.410)	<0.001	2.487 (1.435–4.368)	0.001	2.479 (1.380–4.545)	0.003	2.679 (1.438–5.124)	0.002

* Adjusted analyses were performed by using multivariate logistic regression analyses adjusted for age, sex, primary site, tumor grade, functionality, and liver tumor burden.

**Table 3:** Baseline characteristics of patients with LM-GEP-NETs who received early versus late TAE in unmatched, PS-matched, and IPTW-weighted populations.

	Before Matching				PS Matched				IPTW Adjusted			
	Late TAE	Early TAE	*p*	SMD	Late TAE	Early TAE	*p*	SMD	Late TAE	Early TAE	*p*	SMD
*n*	123	144			123	123			266.69	267.71		
Age (mean (SD))	50.57 (12.42)	51.00 (12.72)	0.780	0.034	50.57 (12.42)	51.10 (12.46)	0.739	0.042	50.77 (12.11)	50.64 (12.49)	0.929	0.011
Male sex (%)	57 (46.3)	64 (44.4)	0.852	0.038	57 (46.3)	56 (45.5)	1	0.016	115.3 (43.3)	118.5 (44.2)	0.878	0.02
Primary site (%)			0.755	0.135			0.947	0.077			0.989	0.045
Pancreas	70 (56.9)	77 (53.5)			70 (56.9)	67 (54.5)			145.1 (54.4)	145.6 (54.4)		
Rectum	35 (28.5)	39 (27.1)			35 (28.5)	37 (30.1)			72.4 (27.2)	75.8 (28.3)		
Small intestine	12 (9.8)	20 (13.9)			12 (9.8)	14 (11.4)			36.0 (13.5)	32.5 (12.1)		
Stomach	6 (4.9)	8 (5.6)			6 (4.9)	5 (4.1)			13.1 (4.9)	13.8 (5.2)		
Grade (%)			0.557	0.132			0.771	0.092			0.912	0.058
Grade 1	15 (12.2)	19 (13.2)			15 (12.2)	15 (12.2)			37.2 (13.9)	32.2 (12.0)		
Grade 2	97 (78.9)	117 (81.2)			97 (78.9)	100 (81.3)			209.9 (78.7)	214.5 (80.1)		
Grade 3	11 (8.9)	8 (5.6)			11 (8.9)	8 (6.5)			19.6 (7.4)	21.0 (7.9)		
Functional tumors (%)	13 (10.6)	26 (18.1)	0.121	0.215	13 (10.6)	15 (12.2)	0.841	0.051	39.9 (15.0)	40.8 (15.3)	0.958	0.008
Primary resected (%)	63 (51.2)	52 (36.1)	0.018	0.308	63 (51.2)	50 (40.7)	0.125	0.213	113.8 (42.7)	116.7 (43.6)	0.889	0.018
Partial liver resection												
(%)	26 (21.1)	17 (11.8)	0.057	0.254	26 (21.1)	17 (13.8)	0.179	0.194	42.4 (15.9)	44.5 (16.6)	0.888	0.019
Liver-directed ablation												
before (%)	19 (15.4)	7 (4.9)	0.007	0.356	19 (15.4)	7 (5.7)	0.023	0.321	25.7 (9.6)	25.0 (9.3)	0.943	0.01
Liver tumor burden (%)			0.168	0.233			0.639	0.121			0.988	0.021
<25%	49 (39.8)	72 (50.0)			49 (39.8)	54 (43.9)			117.9 (44.2)	120.8 (45.1)		
25–50%	31 (25.2)	25 (17.4)			31 (25.2)	25 (20.3)			56.5 (21.2)	56.7 (21.2)		
50%+	43 (35.0)	47 (32.6)			43 (35.0)	44 (35.8)			92.3 (34.6)	90.2 (33.7)		

Abbreviations: LM-GEP-NETs, liver metastases of gastroenteropancreatic neuroendocrine tumors; PS, propensity score; IPTW, Inverse probability of treatment weighting; SMD, standardized mean difference. Late TAE was defined as a latency of TAE ≥4 months after the diagnosis of liver metastases. Early TAE was defined as a latency of TAE <4 months after the diagnosis of liver metastases.

## Data Availability

The dataset is available upon request from the authors.

## List of Abbreviations

GEP-NETs	gastroenteropancreatic neuroendocrine tumors
LM	liver metastases
TAE	trans-arterial embolization
TACE	trans-arterial chemotherapy embolization
RECIST	response evaluation criteria in solid tumors
mRECIST	modified RECIST
OS	overall survival
CR	complete remission
PR	partial remission
SD	stable disease
PD	progressive disease
IPTW	inverse probability of treatment weighting
